# The Elementary Organisms

**DOI:** 10.1007/s10739-024-09773-9

**Published:** 2024-08-16

**Authors:** Ernst Brücke, Daniel Liu

**Affiliations:** Historisches Seminar, Ludwig-Maximilians-Universität München, Geschwister-Scholl-Platz 1, Munich, 80539 Germany

## Abstract

In 1861 the physiologist Ernst Brücke (1819–1892) published “The Elementary Organisms,” calling for a major reform of the definition of the animal cell. An English translation of Brücke’s essay is presented here for the first time. In this translation the numbered footnotes 1–9 are Brücke’s own; alphabetical endnotes A–HH are my own annotations, with additional references to works cited by Brücke. Figures referenced by Brücke but not included in his original essay are also provided. I have also presented an introductory essay to my translation that provides background on Brücke and his arguments: “The Schema and Organization of the Cell,” 10.1007/s10739-024-09774-8, in this same issue of the *Journal of the History of Biology*.

It has been nearly a quarter century since Theodor *Schwann* introduced us to the elementary organisms that form the composite animal body and proved that they transform into the various tissues.^1^ In that well known chapter in his book titled “Theory of the Cells,” he admirably described their importance for the whole organism, bringing about a lasting transformation through a complete series of ideas, effectively taking us into a new era of physiological research.^A^

With regard to the formation of individual cells, he built on *Schleiden’s* claims in a way that has not been fully proven by later experience. He held that cells develop freely in the blastema by molecular aggregation and absorption of liquid. Later research did not confirm this. If such a case of cell-like entities were observed forming in this way, then by our current way of seeing things such an observation would probably be interpreted completely differently. In such a case we would have to conclude that the elementary organisms are not these cells, but rather the molecules that join together to form them.

As for the morphological components of the cell, *Schwann* followed the botanists by recognizing the cell membrane [*Zellenmembran*], the cell contents [*Zelleninhalt*], the nucleus, and the nucleolus.^B^ Yet even back then, this schema of a fluid-filled vesicle with a nucleus and nucleolus could not accommodate all of the parts of all types of cells. Setting aside the fibrous and tubular tissues that are formed by a metamorphosis of cells, there are histological elements [*Gewebtheile*] that are clearly cell-like, but that have a conspicuously more complicated structure than that outlined by our schema. I have in mind the ciliated cells. Should the cilia be considered extensions of the cell membrane, outward protuberances into which the cell contents extend?^C^ This view was obviously not very appealing, and yet the strict schema leaves no space for an alternative. Recently, *Funke* (Fig. 1) and *Kölliker* (Fig. 2) described a structure of the columnar epithelial cells of the intestinal villi in greater detail, which they consider to be a thickened and porous part of the cell membrane.^D^ However, this structure has nothing to do with the cell membrane. Even before I became familiar with its peculiarities, I had determined, based on the behavior of these cells during resorption and from the changes they undergo through the addition of water, that the membrane of these cells is shaped like a cone or a pouch that opens towards the intestinal lumen.^E^*Brettauer* and *Steinach* carried out investigations of these cells in my laboratory, and I have become most decisively convinced that their representation is the correct one (Fig. 3).^F^ The lines of the structure do not come from tubular pores running through a coherent mass. Rather, the appearance of lines is due to the fact that the structure is composed of individual prismatic parts that are not a part of the cell membrane; therefore, I call these structures intestinal rods [*Stäbchenorgan*]. These rods are directly connected to the cellular contents, for if the membrane is detached from the cellular contents, the rods remain attached to the latter, leaving the membrane floating next to them like an empty pouch, completely disconnected from the intestinal rods. Later investigations, parts of which I was able to conduct with far better means of magnification—*Hartnack’s* immersion system No. 10—only served to confirm *Brettauer* and *Steinach*’s results.^G^ Even if every cell was squeezed into this schema, one would not fail to recognize the serious difficulties which the schema presents when it is not only about the cells as such, but about the things which become and emerge from them as well. The histological literature is like a catalog of more or less successful and more or less failed attempts to overcome these difficulties. However, the schema does not apply just to the contiguous tissues, and there are isolated structures for which it is no less important. In their simplest form, spermatozoids are filaments that are thicker and stiffer at one end than at the other; neither membrane nor content, neither nucleus nor nucleolus can be demonstrated in them, not even when the rigid body and the mobile tail are clearly distinguished from each other. On the other hand, if we look at the spermatozoa of a salamander, we find a form so complicated and so different from the usual cell type that we cannot trace it back to the same. We see a fine tip at the front end of an elongated body, which, according to *Czermak’s* illustration of *Salamandra atra* even has a small barb, and on the back end has a tail that carries a thin, fin-like membrane that moves continuously in a delicate, wave-like movement (Fig. 4).^2^ We see all this in structures that we have recognized not as composed of cells, but as the offspring of individual cells.

**Fig. 1:**
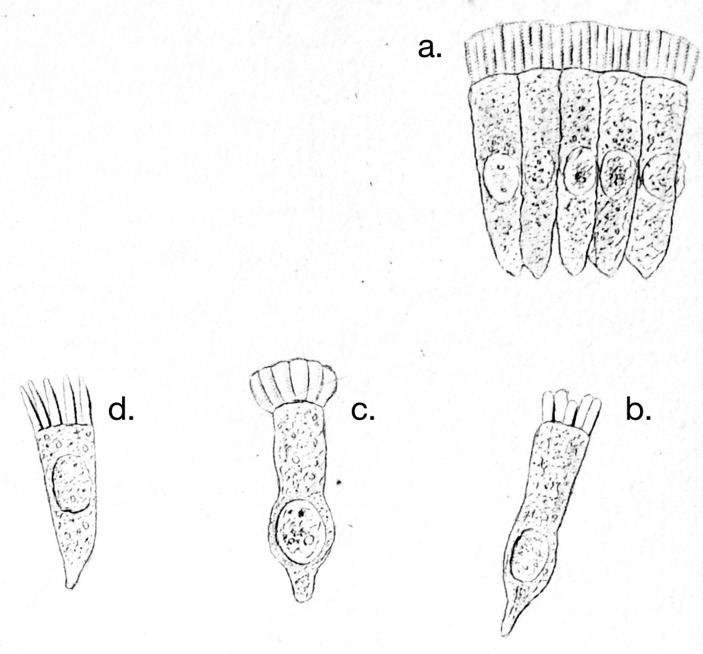
Otto Funke’s (1855) four hypotheses for the structure of the lumen-facing surface of intestinal epithelium cells. Clockwise from the upper right: (**a**) Fünke’s preferred structure, with a thick, porous membrane on the lumen-facing side of the cells; (**b**) as an uneven structure; (**c**) as a single but separate structure from the rest of the cell, capable of swelling; (**d**) as individuated cilia, which Funke dismissed, claiming he rarely observed this

**Fig. 2:**
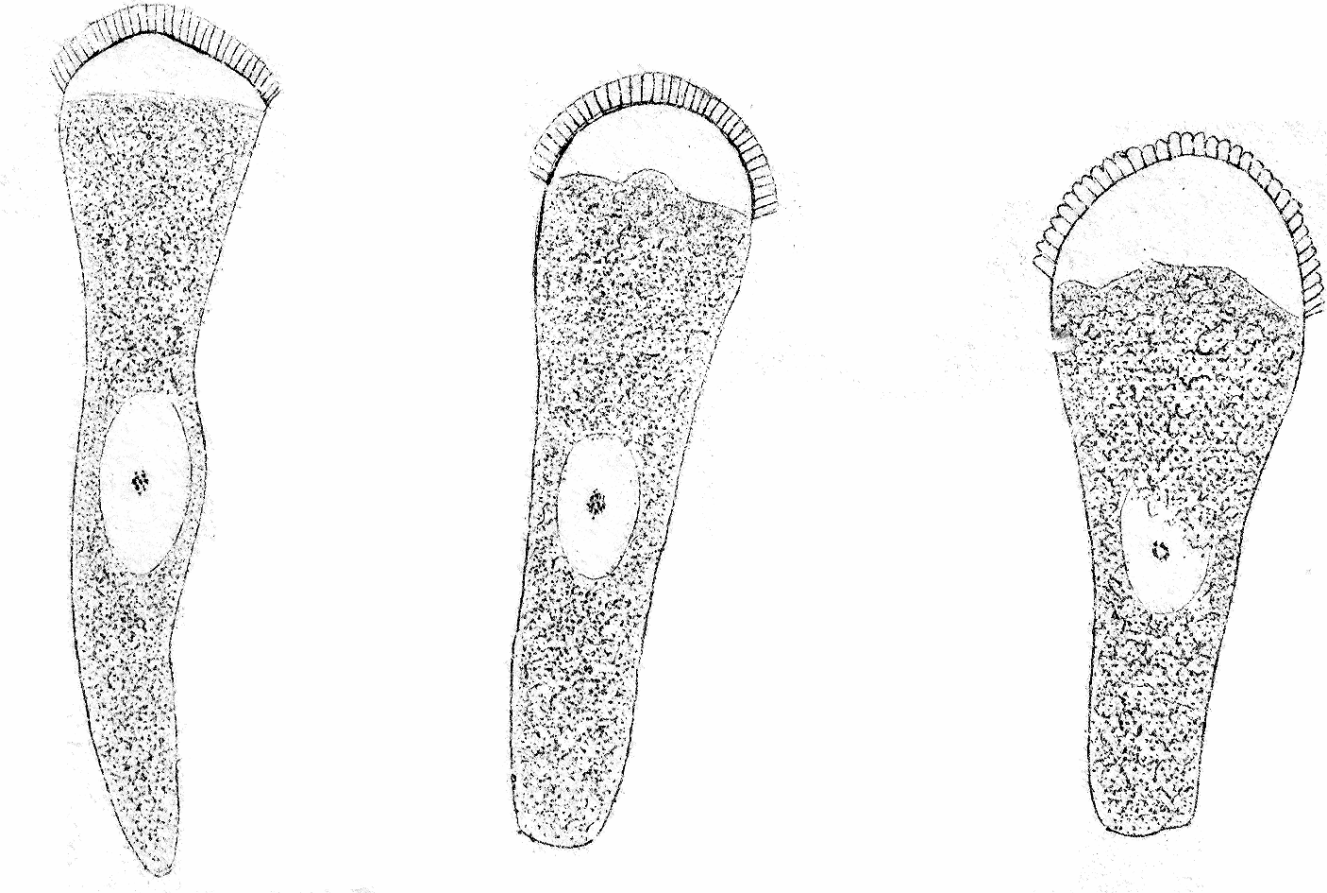
Albert Kölliker’s (1856) theory of the lumen-facing surface of columnar epithelial cells as a thick, coherent membrane. These cells have been swollen by osmosis, showing the porous “striped membrane” as a contour continuous with the rest of the cell membrane

**Fig. 3:**
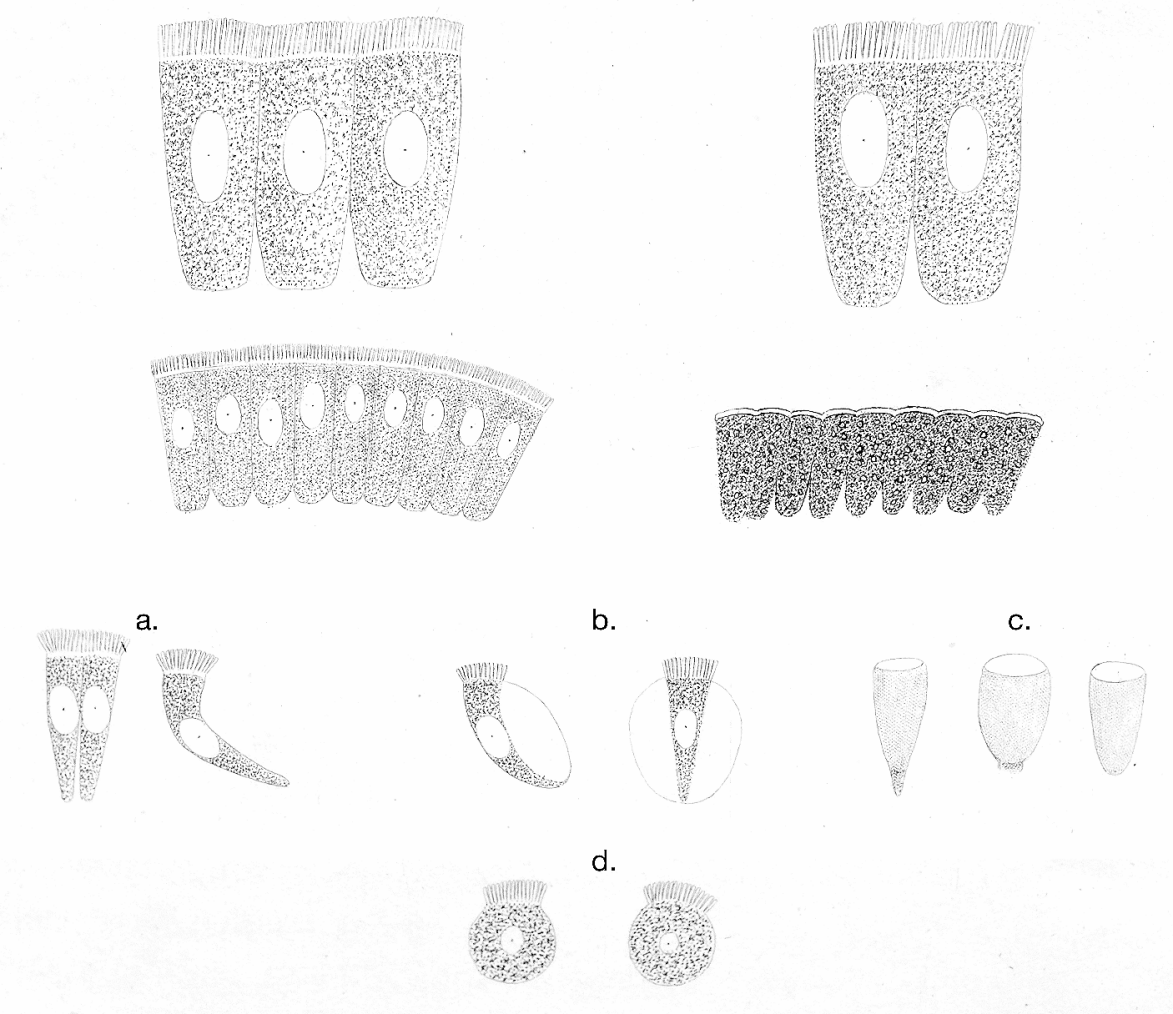
Illustration of columnar epithelial cells by Brücke’s students, Josef Brettauer and Simon Steinach (1857), showing the microvilli as rod-like extensions or “prisms” of the cellular body. **(a–c)** show isolated cells from dog small intestine and placed in water, **(b)** with the cell contents and “rods” of the microvilli remaining a coherent mass and the membrane swollen by osmosis, **(c)** membranes from (b) removed, showing them as open cones rather than enclosing the whole cell. **(d)** isolated cells from rabbit small intestine, swollen with sodium phosphate solution, showing the rods of the microvilli separating to look like “a fine-toothed gear of a pocket watch” (p. 308)

**Fig. 4:**
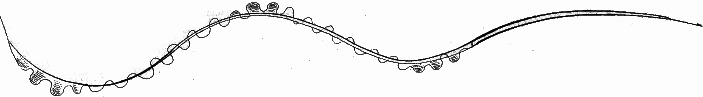
J. N. Czermak’s illustration (1850) of salamander sperm, with a small hook at one tip

What entitles us to believe that our schema exhausts [all the possibilities of] the organization of the cell as such? Do we have grounds to make such an assumption if we cannot recognize any further detail in individual cells, even with our strong magnifications that give us relatively huge retinal images of them? When we were boys catching jellyfish at the beach, holding them in our hands and turning them round and round, what organization could we recognize from the retinal images provided by our naked eyes, which were bigger than the images of cells given to us by the best microscopes? What did we see in them other than a plate-shaped gelatinous glob with some equally gelatinous appendages? Should we be hiding from the fact that various circumstances limit the scope of our microscopic perceptions?

First of all, it is clear that we will not be able to see any objects whose absorbance or refractive index do not differ from those of their surroundings; but we will also miss some objects that do exhibit such differences.^H^

A difference in absorbance must be *considerable* for an object to become visible, because only then will there be a noticeable difference in light and color, given the extraordinary thinness of the layers they pass through. Such *considerable* differences occur in fact quite frequently in particular parts of cells: we call these pigment granules or pigment masses, names that mean nothing other than that these parts differ substantially from the remaining materials in the cell in their ability to absorb light.

Furthermore, this material’s absorbance is so uniform that it does not provide us with any aid for identification: because of the extraordinary thinness of the aforementioned layers that the light has to travel through, only very considerable differences can have an effect, while small ones are completely imperceptible.

The essential basis for all microscopic differentiation is always the difference of the refractive index, in that this difference causes refraction and reflection. I am deliberately omitting diffraction, since this can only be perceived under circumstances in which our powers of microscopic discernment are no longer reliable. However, the clarity of the phenomena of refraction and reflection essentially depends on the degree of the difference of the refractive indices of two adjacent media, among other things. This difference, even when undeniably present, can be so small that forms and dimensions are no longer visible, even though they would not have escaped us had there been a greater difference in their refractive indices. Perhaps every microscopist will have sufficiently convinced himself of this when infiltrating microscopic preparations with liquids of different optical density. Add to this the indistinctness caused by the superimposition of masses that are irregularly shaped and differently refracting, and we will see why we cannot be very sure of details revealed even in large images. Finally, the dimensions themselves set limits to our perceptions, at least for now, insofar as our microscopes are still not mature enough, as they will be forever, and insofar as the action of the microscope depends on which physical parts interact with the waves of light.

I cannot imagine that there is any microscopist who seriously believes that our microscope images give even an approximately complete overview of the structure of cells, and if it is said that the cell membrane is structureless, or that the protoplasm is a homogeneous mass, etc., then this should probably mean nothing else than: the cell membrane appears structureless to us, the protoplasm appears to us as a homogeneous mass. If one wanted to use these expressions in a stricter sense, it would betray our colleague’s limited horizons in a way in which I would not like to presuppose.

As commendable as it is to adhere strictly to what is directly observed, it is necessary not to close the mind’s eye to what is inaccessible to observation. We should thus not overestimate the value of our microscopic perceptions and build up physiological doctrines with the help of keywords like “cell membrane,” “cell contents,” and “cell nucleus,” terms which a future generation might rather reject.

Let us first ask ourselves what we can infer about the cell’s finer structure that is inaccessible to direct observation. Structure—if it is understood as nothing more than a particular type of arrangement of the smallest parts that, when a body expands through heat, change their position but not their size—structure in this sense surely belongs to all chemically compounded bodies. We cannot deny this fact even in the case of bodies that we regard as being chemically simple, for it is possible that some of them differ from each other only by the way in which the smallest parts are combined into larger groups, or that they differ from one another only by the structure of the molecule which we have hitherto mistakenly regarded as their atom. We know that the structure of the molecules of the organic substances that make up the cell’s composition is very complicated; their high atomic weight shows that they are made up of numerous building blocks [*Bausteine*]. But for the cell we cannot be satisfied even with such a complicated molecular structure. We cannot conceive of a living, vegetating cell with a homogeneous nucleus and homogeneous membrane and a mere protein solution for its contents, because those phenomena that we call the vital phenomena [*Lebenserscheinungen*] we do not perceive in protein as such. We must therefore attribute to the living a different degree and kind of structural complexity, apart from the molecular structure of the organic compounds which they contain, and this is what we call *organization.*

The composite molecules of organic compounds are workpieces that are artfully [*kunstreich*] joined together to form a living structure, not piled up uniformly, one next to the other.

We not only see that the cells grow, increasing their volume by absorbing foreign substances, but we also perceive a variety of other activities in them: one of them moves continuously; another changes its shape by twitching in response to a stimulus; a third emits impulses which, carried through living wires, exert their effect in distant regions of the organism.

In composite organisms we see different effects emanating from different parts, which we call organs and systems of the body, and we can hardly think otherwise than that, in the cell as well, different effects emanate from differently constituted, differently built parts.

Of course, we would not expect a recapitulation of the organs and systems that we find in the human organism; we know that this is not the case even in the lower animals, we know that as dimensions decrease the means by which the forces of the inorganic world are harnessed in the organism changes. But apart from these differences and apart from the smaller number of bodily parts, we have no right to consider one of these small organisms to be less artfully constructed than one of larger dimensions. We are aware of this not only as we investigate the smallest animals, but also as we study cells both in animals as well as in plants. We must always see in the cell a small animal body, and we must never lose sight of the analogies that exist between it and the smallest animal forms.

The resemblance between an amoeba and a crustacean blood cell, between an infusorian and a spermatozoid or a detached ciliated cell could, in isolation, be regarded as something superficial or accidental; but the unicellular plants show the direct connection between free-living organisms and those that can only exist as integrated parts of a larger whole.

This is the point of view to which I felt I had to lead the reader before I begin to discuss some questions whose final answers ought to be achieved through many dispassionate discussions, unrestricted by any traditional prejudices.

## The Cell Membrane^I^

It is now generally accepted that the cellulose membrane [*Cellulosemembran*] of the plant cell is not analogous to the membrane of the animal cell. Like the calcium carbonate shell of a snail, the cellulose membrane is the plant cell’s house and later its coffin; the membrane [*Membran*] of the animal cell is initially its skin [*Haut*]. This leads us to the question of whether or not a membrane is a necessary attribute of the animal cell. If the skin is understood as nothing more than the outermost layer, without demanding that it should be distinctly different in consistency or composition from the one below it, then there is nothing against attributing such a skin to every cell. This is to say nothing other than the generally recognized truth, that the surface of every limited body can be distinguished from its interior. If instead we demand that this skin has a considerably greater solidity to lend coherence and protection to those [layers] below it—as ought to be the case to justify the term membrane—then I must completely agree with the opinion of Max *Schultze*, that such a membrane is not a necessary attribute of the cell, and is probably not even generally present when it is young, but rather where it is found it has formed only later through a gradual process of compaction and hardening.^3^

**Fig. 5:**
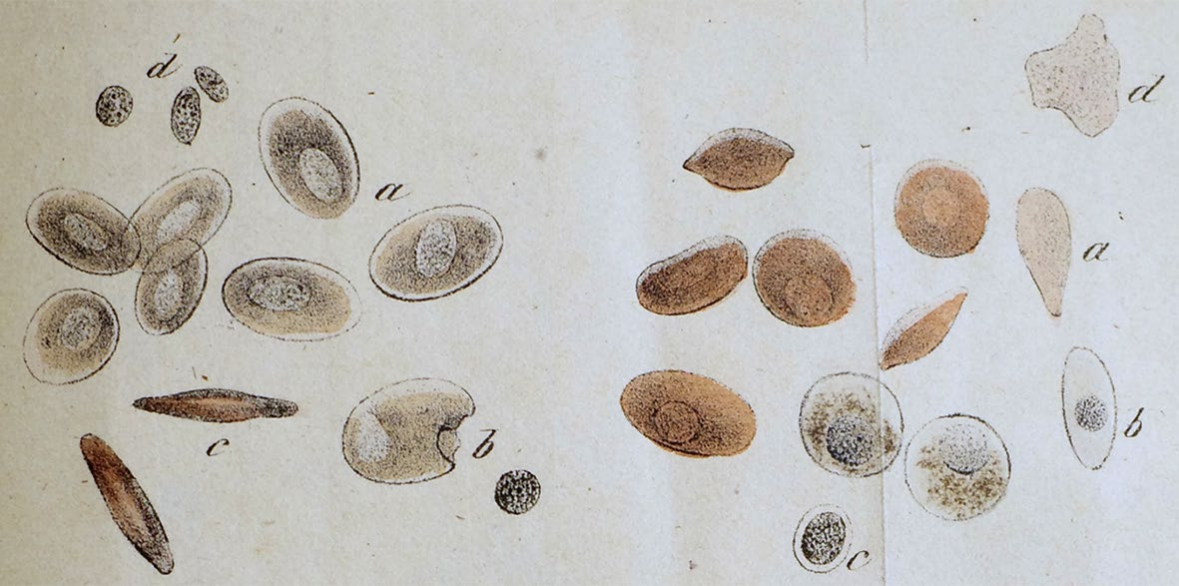
C. H. Schultz’s (1836) illustrations of nucleated salamander blood cells. On the left: normal blood cells, including one (**b**) with its nucleus removed. On the right: blood cells swollen in water, **b** showing the nucleus “rolling around” inside the membrane, **d** showing the blood cell after it has burst open and lost its nucleus and coloring

Wherever a membrane is assumed, it must be proven. Such proof must not be taken lightly, in confidence that the dogmas of cell theory are correct, and the means of proof must be subjected to careful criticism. One of these assumptions has always been the behavior of cells towards water, and C. H. *Schultz* and *Schwann* have used this to prove that the blood corpuscles possess such a membrane (Fig. 5).^J^*Schwann* states:

C. H. *Schultz* was the first who proved the blood-corpuscles to be vesicles [*Bläschen*]. He relied especially upon the manner in which they were acted on by water, whereby they lose their colouring matter, swell, and become round, and under which circumstances he frequently saw the nucleus roll about within the round and very transparent vesicle. The last fact would of itself be sufficiently conclusive. I have not as yet observed this fact; on the contrary, in most instances the nucleus decidedly adheres to the internal surface of the wall of the vesicle, eccentrical as in all cells, though it may probably also sometimes become detached. The fact, however, of the blood-corpuscles becoming swollen and round, renders their cellular nature highly probable. If the envelope [*Hülle*] of the blood-corpuscle were not a flattened vesicle, it might indeed lose its colour and swell in water, but it would retain its flat form, like a sponge when filling with fluid.^4^

I have never seen a nucleus rolling about in a blood corpuscle, and I believe most of my colleagues will agree with my opinion that this statement is based on an illusion.^K^ For me only the last reason given by *Schwann* has significance, but even this is not absolutely well founded. It is known that different organic structures swell up differently in different directions due to their finer structure, and this could also be the case for blood corpuscles. The fact that the diameter of blood corpuscles when they form spheres in water is smaller than the largest diameter of the disk from which the sphere was formed—even this is not proof of the existence of the membrane. This, too, could be a consequence of finer structural conditions. For example, instead of a single disk, imagine a disk-shaped system of very many similar disk-shaped vesicles connected to each other in rows and layers: this would also become spherical by swelling in an analogous way. Even if the existence of a solid outer envelope was admitted, then its response to water still does not at all prove what it is supposed to prove: namely that the blood corpuscles are vesicles whose content is liquid apart from the nucleus. If, instead of a liquid, a soft, absorbent substance was surrounded by a more solid shell, then the phenomenon might be outwardly the same. In this substance capable of imbibition, manifold structural relationships could be present without externally changing the result of the swelling process. The vesicular nature of the blood has been taught for a fairly long time, and it must be admitted that this unanimity was due more to the silence of its opponents than to the force of the defenders’ arguments.

As little as the swelling observed in blood corpuscles demonstrates their vesicular nature or even suffices to prove the existence of their membrane, neither can analogous phenomena in other cells be used for the same purpose. Wrinkling [*Faltenbildung*] has been another means of identifying the cell membrane. Yet wrinkling never proves that there is a solid envelope surrounding fluid contents, but only that the reagent used makes the interior of the cell shrink more than the outer or outermost layer of it. Admittedly, this is generally due to the greater cohesiveness of the outer parts. But this does not mean that this cohesiveness does not extend deeper in places, or that the appearance of wrinkles, rather than being a mere skin, is not instead the main mass of the whole cell body [*Zellenleib*], collapsing around one or several cavities or watery, soft structures. Finally, one must be careful about whether the greater consistency of the outer layer is not created by the effect of the reagent.

A third, likewise only conditional means is provided by molecular movement.^L^ As proof for the cellular nature of pigment cells, *Schwann* already cited the fact that a molecular movement can already be perceived within the cell, similar to that displayed by free pigment grains in water.^M^ However, not every kind of movement of small granules inside a cell indicates that the cell is a vesicle with liquid contents. First of all, granular movements can be delimited by canals or cavities in the cellular body, which are not general cell cavities. Second, granules can move in connection with other moving parts of the cellular body.

The most beautiful molecular movement that I have seen in cells of the human body is visible in the salivary corpuscles.^N^ Yet I have not yet been able to convince myself that these are hollow vesicles with fluid contents: for when they are squeezed, the granules do not flow out. The whole body is compressed into a flat cake in which the granules remain motionless. Even if the cover glass is lifted and more liquid is allowed in again, molecular movement does not restart in such squashed salivary corpuscles.

Some attach great importance to what is called the lifting of the cell membrane upon the addition of water. The water is said to penetrate between the contents and membrane, eventually inflating and bulging out the latter in the form of a bubble. Of all uncertain shibboleths this seems to me the most uncertain. It is known that droplet-like formations sometimes emerge from cells upon adding water, creating the appearance of a lifted cell membrane that is so beguiling that even very famous and excellent microscopists have allowed themselves to be misled by it. Even a double outline on a bulge formed by the addition of water cannot prove the presence of a membrane; for it is known that some animal structures form so-called vacuoles on the addition of water, i.e., that the water accumulates in individual spaces in the interior in the form of droplets, and by its accumulation drives apart the surrounding substance, which gives the impression of a viscous mass.^O^ Such a vacuole only needs to occur at the edge and be covered by a very thin layer of plasma to give the appearance of a detached membrane.

Obviously, the surest way to convince oneself of the existence of the cell membrane is to isolate the membrane completely. As far as I know however, this has been successful only in one type of cell without injuring the contents, the columnar epithelium. Yet it is precisely in these cells, as *Brettauer* and *Steinach* have shown in their treatise, that the membrane does not surround the whole cell evenly, but only forms a cone-shaped mantle around it, and this is the only reason why it is possible to isolate [this] cell membrane without mechanical injury to the contents.

Emptying the membrane has been demonstrated several times by squeezing the cell, by *Purkyně* and *Raschkow* on the squamous epithelium [*Pflasterepithelium*] and by *Schwann* on the cartilage cell.^5^ Without denying the existence of the membrane in more developed cells of this type, I cannot attribute complete validity to these demonstrations under all circumstances, because fluid will be squeezed out of every cellular body that is squeezed: it is often very difficult to judge with any degree of certainty whether what remains is a bare cell membrane, or rather if it is instead the contiguous, main mass of solid parts distributed in different regions of the cell body.

The last way of convincing oneself of the existence of the cell membrane is to recognize its outline in the intact cell. But this raises other issues. The membrane is not only distinct from the cell contents in its density, but it also must have a certain thickness, for a simple contour line in and of itself cannot establish the diagnosis of a membrane. There must always be two contour lines, one representing the outer boundary of the membrane, the other the inner.^P^

Some microscopists have concluded that there is a membrane from just one contour line, apparently on the premise that the cell content is a *fluid* whose refractive index differs little from the surrounding medium. This is completely impermissible under the premise which I have sought to establish above, namely, that the cell content is from the outset a *structure* of solid and liquid parts. The difference in density between the cell contents and that of the surrounding medium is a sufficient basis for the *one* contour line, even without an enveloping membrane. It is through the second outline that a difference in density between the outer envelope and the contents can be recognized. It goes without saying that the magnification used must not be driven beyond the real power of the instrument by strong oculars, because this will produce a second outline that has no basis in the nature of the cell, but rather only in errors of the optical apparatus. Therefore, only cell membranes of a certain thickness can be recognized in this way.

Now if we are to speak of thick cell membranes, we must first clarify what we mean by thickened cell membranes and by intercellular substance. For plants the definition of the thickened cell membrane is easy, since the cellulose membrane itself must be regarded as an excretion that is completely distinct from the original cellular body and is located outside of it, and whose thickening is merely the result of the accretion of differently composed secondary layers.^Q^ This is not the case with animal cells. There is no membrane that can be distinguished against the cell as such. The former is a part of the latter, and if it becomes thicker, this happens either by growing like other parts of the cell, or by new parts of the cell body being drawn into the hardening process by which it itself was formed. This hardening process seems to me to be directly related to the formation of certain so-called intercellular substances.

I find that the theory of intercellular substances is an erroneous one, at least in the way it is usually presented. My view of its development closely follows that of Max *Schultze* in his aforementioned treatise. In order to justify my views, I must first point out the two basic errors under which the questionable doctrine arose. The first is that of exogenous free cell formation.^R^ The cartilage cells were held to develop freely in the intercellular substance, and thus the intercellular substance had to be regarded not only as something different from the cartilage cells, but also as something partially existing before them.^S^ Secondly, it was held that the cell membrane formed earlier than the cell content and therefore surrounded it from the beginning; therefore, what lay outside of it could no longer belong to the cell, and was therefore given the name intercellular substance.

We know in particular from observations of cartilage that newly formed cells lie initially one next to the other.^T^ To return to the doctrine of exogenous free cell formation: we would also have to assume that intercellular substance subsequently flows in between these newly formed cells from elsewhere and pushes them apart. The intercellular substance would thus manifest as a formation without organic structure, passing from the liquid to the solid state through a kind of thickening and coagulation process. We have no justification to make such an assumption, because we do not yet know of any kind of organization which builds itself up in the animal body independently of the cells. I find it much more likely that the formation of the intercellular substance therefore comes from the cells, and there would be no obstacle to this assumption if we no longer assume that the cell membrane is formed before the cell body. Let us imagine that the outermost layer of every cartilage cell transforms through steady growth in a substance which we will call “cartilage substance” in the narrower sense of the term, and that in doing so it bonds to the same layers of neighboring cells so that their borders can no longer be determined, creating the intercellular substance, as it has been identified under the microscope. If the part of the cell body that is not involved in this metamorphosis is still surrounded by its own, differently refracting layer, cell membrane, or cartilage cell capsule, then this is a secondary formation that forms the basis of either the already metamorphosed or the not yet metamorphosed part.^U^ According to what I have observed so far in the development of the cartilage, the former seems to me more probable. It seems to me that in this kind of development of intercellular substance, the layer surrounding the non-metamorphosed part of the cell body becomes firmer than the rest, so that the capsule is formed by differentiation and becomes visible by its inner and outer outline, and can possibly detach itself from the surroundings to such an extent that it is possible to isolate it by mechanical force. The accumulation of free fluid within the cartilage cells, as already observed by *Schwann*, I also consider to be the result of a secondary metamorphosis.^6^

**Fig. 6:**
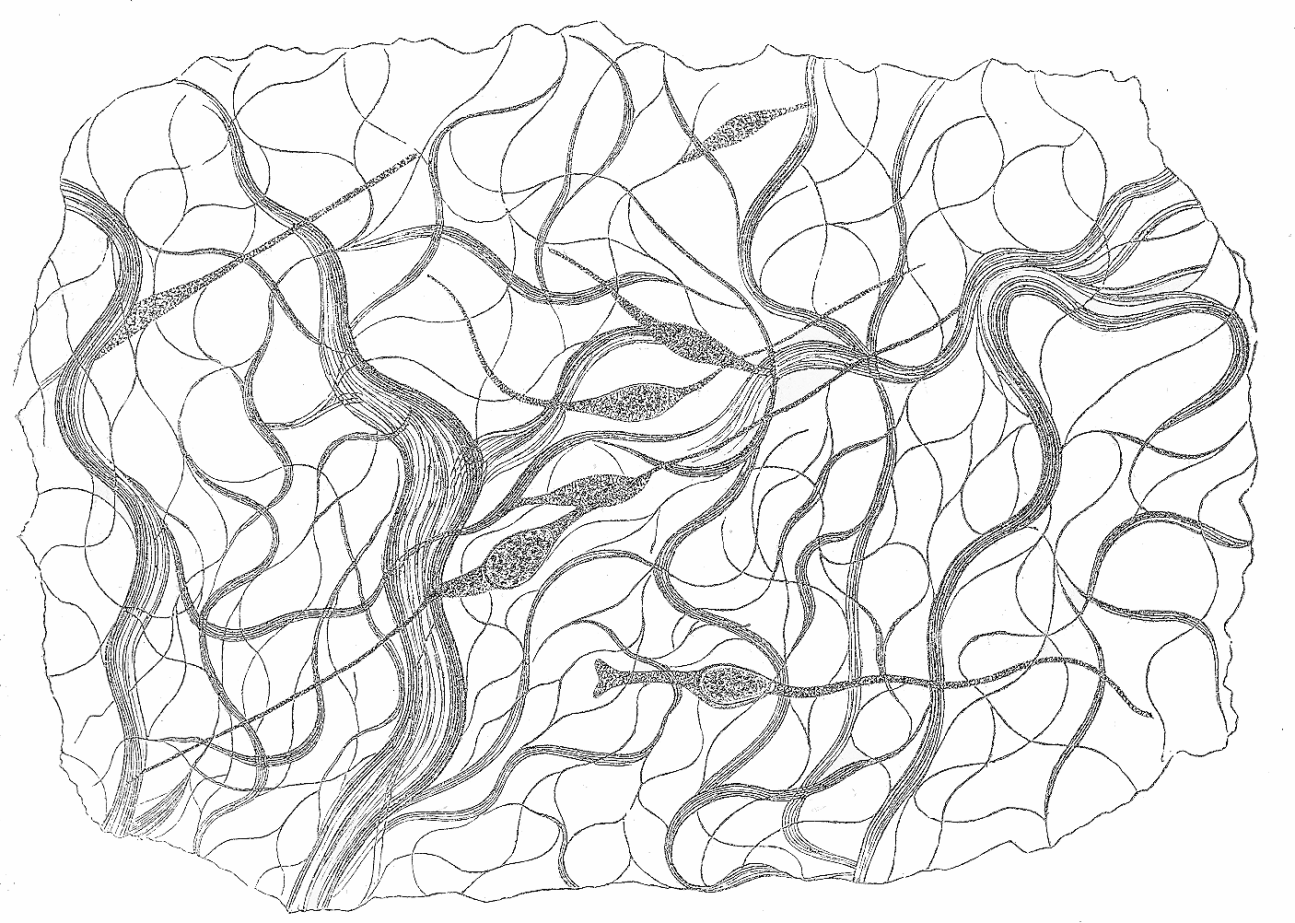
Schematic illustration of embryonic connective tissue by Alexander Rollett (in Stricker 1870, p. 88), from serous membrane of a five month old human embryo, showing the nucleated, spindle shaped *Bindegewebskörperchen* as precursors to fibrous connective tissue

If this view of the development of intercellular substance is accepted as correct, then I believe a similar understanding can be reached in the well-known controversy over connective tissue development, as Max *Schultze* has already pointed out. I have never been able to convince myself that fibrous connective tissues might develop between cells from an intercellular substance that is both different and foreign to them; the images I have obtained of developing tendons lead me to the exact opposite view. I am basing myself here not only on my own investigations, but also on those of Dr. *Rollett*.^V^ Years ago he was investigating connective tissue, and he showed me a preparation taken from a developing chick in the egg: the connection of the fibers to the cells from which they emerged and with their nuclei could be followed unambiguously (Fig. 6). Then again, it cannot be denied that the cartilage’s intercellular substance is both directly connected and immediately transitions to the connective tissue. But we can easily explain this if we consider intercellular substance as a product that originally emerged from cells. Again, I consider Virchow’s connective tissue corpuscles [*Bindegewebskörperchen*] with their nuclei to be the part of the cell body that has not been included in collagenous metamorphosis.^W^ These are the connective tissue corpuscles, whose analogy to the bone corpuscles [*Knochenkörperchen*] can be demonstrated beyond doubt; however, we must reject this analogy for other forms that have nevertheless been categorized as connective tissue corpuscles. Among the latter are: cells with extensions which transform into elastic fibers; cells with extensions which have no demonstrable connection with the development of the collagenous substance, and which either retain their shape or whose further metamorphosis is unknown; finally, branched cavities whose origin from cells is not proven, tissue lacunae. The development of secondary bone is quite similar to that of the connective tissue, except that here neither common connective tissue nor fibrous tissue is formed, since the collagenous substance is immediately transformed into bone by the incorporation of calcium phosphate. The analogy between cartilage corpuscles [*Knorpelkörperchen*], bone corpuscles, and connective tissue corpuscles thus remains completely valid. Likewise, everything Virchow taught about the role of these corpuscles in pathological processes remains valid and seems perfectly understandable to us if we take into account that a capacity for reproduction only remains in that part of the cell that still constitutes an organism, analogous to the original embryonic cell.

Thus far, I have assumed that the collagenous and cartilaginous substances are formed by metamorphosis of a part of the cell body. However, I would not be able to convincingly refute any claim that they instead arise from the surface of the cell body as new growth. With the human organism it is generally easy to use common language to define what “metamorphosis” or “new growth” are, because we have studied the structure of the human body down to its elementary parts. But the situation is different with the cell, whose structural relations elude our means of magnification down to its basic features. How would we determine in what form the cell assimilates the collagenous and cartilaginous substances, or in what form they release them? I believe the cellulose membrane of the plant cell is a new growth because it consists of a material that differs fundamentally from that of the cell body. However, this is not necessarily the case for the animal cell membrane and the so-called intercellular substances aforementioned, because although the collagenous and cartilaginous substances are chemically different from the main mass of the cell body, this difference is not so great that we must therefore consider it impossible for one substance to be gradually converted into the other by the absorption and release of certain materials. For me the determinative factor was the fact that only the nucleus and a very small part of the cellular body remains, especially in tendon development. This can admittedly be understood as atrophy as well, but, in general, since growth happened and one part decreased while the other increased, I think it is more natural to assume that the increase of the latter took place at the expense of the former. Whatever one thinks about this, it will probably have no practical consequences for quite some time. However, it is essential to decide whether the collagenous and cartilaginous substances are regarded as having either originated outside of the cells and thus preexisted them, or whether they should be regarded as a product of the cells that takes part in their vital phenomena, either for a while or permanently.

## Nucleus and Nucleoli

Although it is no longer universally accepted that the nucleus forms the cell in the way that *Schleiden* and *Schwann* described, histologists still broadly accept the proposition that every cell must have had a nucleus at least in its early life. Even those who admit that this cannot be proven for human and mammalian blood cells are nevertheless of the opinion that at least every cell capable of production [*productionsfähige Zelle*], every cell capable of generating another, has a nucleus.^X^

But we are dealing not just with animal cells but plant cells as well, and we have to take these into consideration. If we are adhering strictly to observation, then it follows that all cells of the phanerogamous plants have nuclei when they are young, but among the cryptogams there are cells both with and without nuclei.^Y^ Furthermore, a cell’s generative capacity cannot be dependent on the presence of nuclei, since multiplication by both division and budding has been observed in unnucleated cells. We must admittedly keep in mind that the nucleus could have a refractive index that is very close to that of the cell contents, thereby escaping observation; but this alone gives us no reason to assume its existence where it is not seen, as long as its necessity is not proven for other reasons. In my opinion, so long as this has not happened *it is not justified to include the nucleus as an essential and necessary component in the conceptualized schema for the elementary organism*.

During the multiplication of cells by division, if the new cell is to get a nucleus there is sometimes an opportunity to see how the nucleus of the old cell divides first, even before the cell’s remaining mass separates into two halves.^Z^ In the case of endogenous free cell formation, the nuclei of the daughter cells are seen first, and this type of cell reproduction has never been observed in non-nucleated cells. Some have thus found reason to give the nuclei a special reproductive function in cells, and some believe this has been confirmed by *Balbiani’s* discoveries in the infusoria, in which he recognized the nucleus as an ovary and the nucleolus as a testis.^AA^ However this analogy, which is in fact what we find here, finds its limits upon closer examination. *Balbiani* has only ever observed mutual fertilization by mating, and never self-fertilization or fertilization of the ovary of an individual by the testis of the same individual. Apart from the familiar fertilization phenomena whose products are whole composite organisms, we lack any evidence to assume this kind of fertilization in cells. For our physiological aims we cannot rely on the morphological similarity of the infusorial nucleus to the cell nucleus.

The belief that the nucleus plays an important role in reproduction cannot be said to be incorrect; however, this belief has not been made so probable as to justify its current status as a general principle.

What would be the objections to the claim that the nucleus is completely passive in every kind of reproduction: division, budding, and endogenous cell formation?^7^ There are numerous examples in which division takes place without intervention of nuclei. In these cases, division must start from the protoplasm itself (or from the protoplasm and primordial utricle [*Primordialschlauch*], for those who assume a primordial utricle).^BB^ But why should this not also be true when nuclear division takes place? We could just as well assume that the protoplasm presses inward and cuts the nuclear mass into two or more parts, even before a division of the cell body becomes visible externally. It cannot be argued that the soft protoplasm cannot constrict and pinch off the hard nucleus: first, it is well known that one must be very careful with such assertions where vegetation phenomena [*Vegetationserscheinungen*] are concerned; and second, it cannot be proven that the nucleus is hard at the time when it divides. One could imagine a loosely held together nucleus dividing itself, and just as easily imagine it being divided. It is an entirely different matter with the consistency of the nucleus.^CC^ The cell theory holds that the nucleus is the first solid element of the cell, even though this has never been proven. One cannot discount the idea that the nucleus is by nature a very soft mass, a mass of lower consistency than the protoplasm, and that it hardens only later, either on its surface (vesicular nucleus) or in its whole mass (solid nucleus). Against such a claim it cannot be argued that the nucleus is firm even in young cells, because generally speaking its firmness is still lower than in old cells; and, at the same time, although the nucleus is found in young cells rather generally, one cannot also then claim that a cell is so young that its nucleus has not already hardened because it was originally soft. On the other hand, every other view could be put forth for all manner of probable reasons, e.g.:

1. That the bright spheres that form during furrowing, and which are the mothers of all animal cell nuclei, are apparently very soft, and that only after the completion of furrowing do they harden as nuclei of the germinal cells,
2. That nuclei, particularly in younger cells, refract light more weakly than the surrounding protoplasm, and thus probably also contain fewer solid components than the latter,
3. That there are cell nuclei that still have a soft, loose, droplet-like quality when they are fully mature, e.g., in the motile pulvini of *Mimosa pudica*.

I mention these things to point out that views diametrically opposed to the currently acceptable ones can be made to seem at least as plausible.

Whoever claims that the nucleus behaves passively during reproduction could again refer to the fact that budding takes place completely without a (visible) nucleus, e.g., in brewer’s yeast. And finally, it could be rightly said that there is not a single fact which proves an active participation of the nucleus of the mother cell in endogenous cell formation.^8^

Let us mention one more dogma of cell theory, namely that the nucleus is the first part of the daughter cell that is formed—and let us convince ourselves that this, too, cannot be proven. While it is true that the nuclei are the first thing that is perceived of the daughter cells, does this also prove that it is the first thing that is formed? The nuclei lie embedded in the protoplasm, and who can say that the body of the daughter cell in its first embryonic state, long before a cell membrane is even created, can be distinguished from the body of the mother cell by our optical instruments? Consider the following process: The first rudiment [*Anlage*] of the daughter cell is a small mass of protoplasm that cannot be distinguished from the protoplasmic mass of the mother cell with our instruments. This mass expands and forms a cavity in its center, against which it is then separated by a membrane, and sooner or later one or more visible corpuscles are formed within it. The cavity with corpuscles would be the nucleus with nucleoli. This now grows, while the surrounding thin protoplasmic layer—the actual first rudiment of the daughter cell—still cannot be distinguished from the protoplasm of the mother cell, because both touch each other directly and are not separated from each other by any other refractive boundary layer. Finally, the cell membrane forms, visibly separating both from each other. Would this not result in same series of microscopic images that have been observed and interpreted in the completely opposite way? I believe that those who do not rely on the testimony of others but observe for themselves without prejudice, will agree with me that we have no positive knowledge of either the origin or the function of the nucleus, indeed that even the constancy of its existence appears subject to essential limitations, if one takes the cells of the cryptogams into consideration and does not assume from the outset that the nucleus must still be present even where it is not seen.

## The Cell Contents

It seems to me that it is our beliefs regarding the contents of the cell that will have to make the furthest departure away from the original principles of cell theory. The cell contents were originally held to be a fluid that accumulated between nucleus and membrane. For us, the cell content is the main mass of the cellular body itself, a complicated structure of solid and liquid parts. Since we do not recognize the cell content as a fluid, if we are then asked whether we believe it is solid, we answer: No. And if we are asked whether it is fluid after all, we answer again: No. The terms “solid” and “fluid” as they apply in physics do not entirely apply to the entities we are dealing with here.

I cannot compare the cellular body’s state of aggregation with that of iron, lead or sulfur in the solid state, nor with that of these bodies in the liquid state. The question of whether the living cell body is solid or liquid is fundamentally as absurd as if I wanted to ask whether the body of a jellyfish or a snail is solid or liquid, in the sense that physics attaches to these terms. Even our terms for the so-called mixed states of aggregation are inadequate. If we were to say that the cellular content is a slimy, gelatinous, or slushy [*sulzig*] mass, this would be no better than if someone knowing nothing about the organization of the medusae described them as gelatinous masses or as living jelly.

If we consider the vital phenomena that we perceive in the cellular content, then we are necessarily led to recognize a relatively complicated structure in it as well. Let us first focus on just one point: the phenomena of movement.

It is sufficiently proven and generally accepted that the contractile substance of striated muscle originates from the cellular contents. Even with our imperfect instruments we have already recognized a rather complicated structure in this contractile substance. From observations made in ordinary and polarized light, and especially from the invariability of their optical properties during contraction, we have concluded that both the fibrils and the *Bowman’s discs* are made of *sarcous elements*, which in turn are composed of fluid and exceptionally many smaller bodies that I have suggested calling disdiaclasts.^DD^

**Fig. 7:**
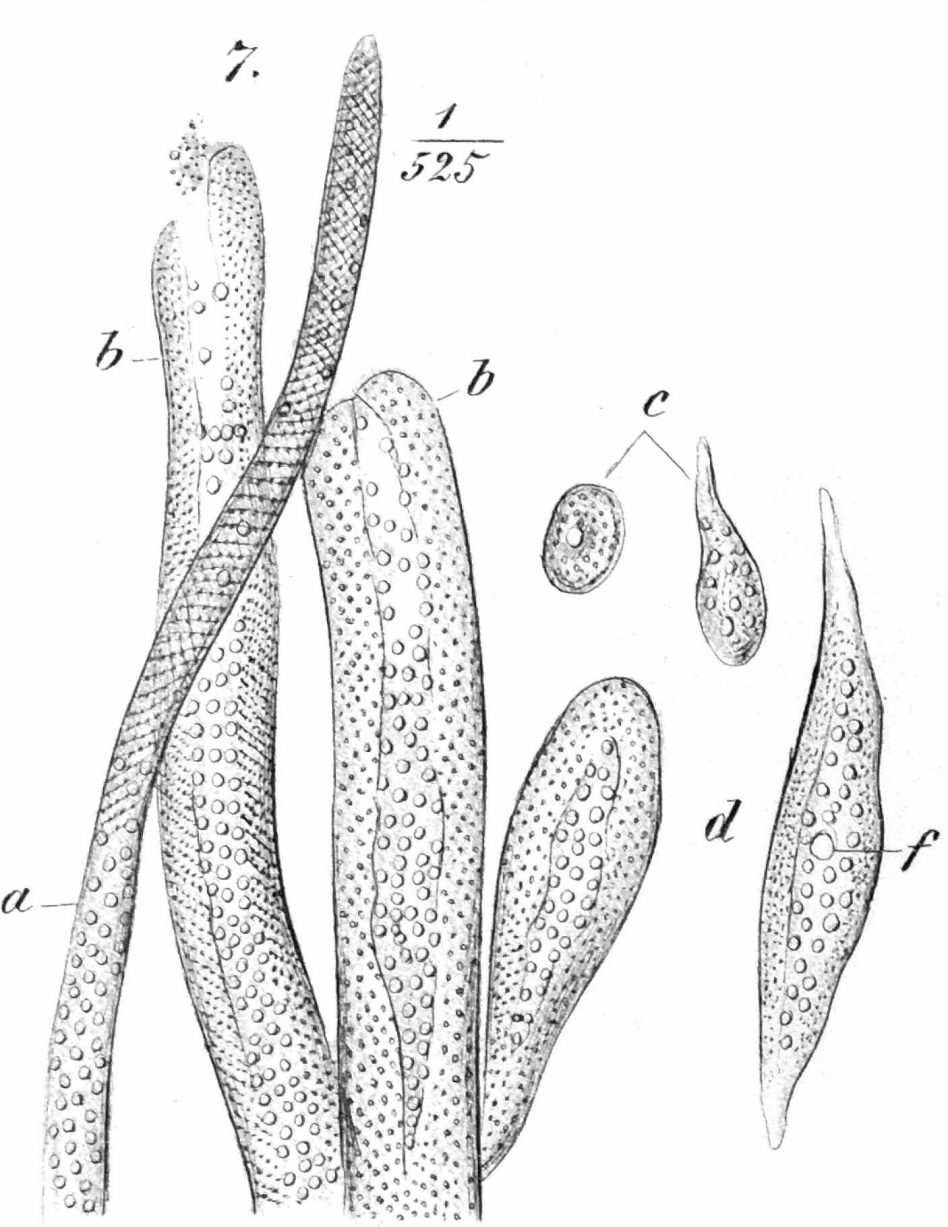
Tivadar Margó’s (1860) image of octopus muscle structure. The small, round shapes are the birefringent *sarcous elements*, corresponding to the banded sarcomeres in vertebrate muscle

**Fig. 8:**
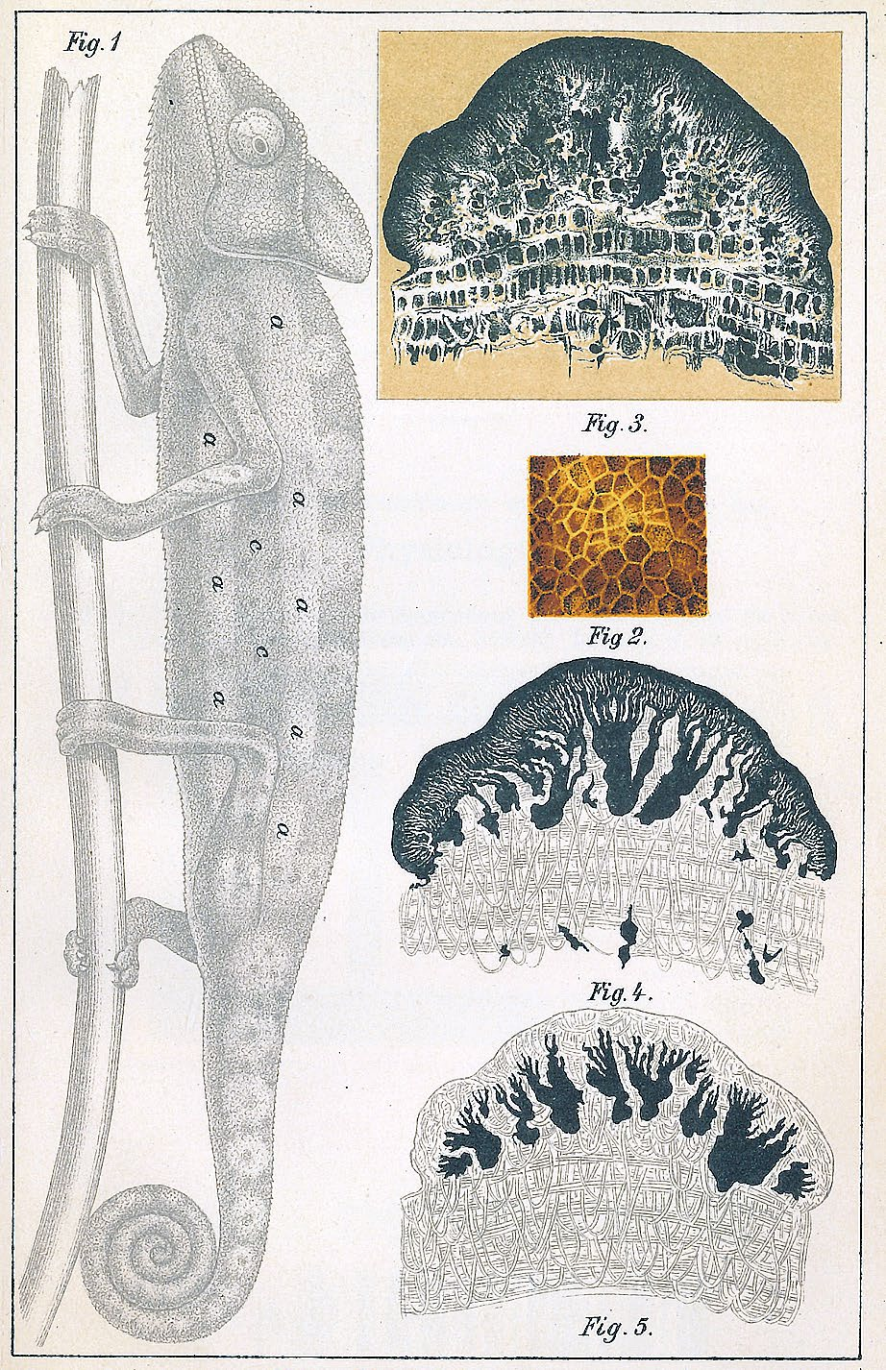
Plate from Ernst Brücke’s (1852) study of chameleon skin (rearranged and reprinted in 1893 in *Ostwald’s Klassiker der exakten Wissenschaften*). The branched chromatophore cells are shown in various states correlating to the chameleon’s changing color

*Margo’s* investigations of bivalve adductor muscles have further shown that muscles hitherto regarded as smooth have proven under greater magnification to be both transversely striated and also to contain *sarcous elements*; these are much smaller than those found in vertebrate skeletal muscle but are otherwise the same (Fig. 7).^EE^ It is reasonable to assume that the same applies to all other so-called smooth muscle fibers or contractile fiber cells. The contractility phenomena here are relatively simple, and at a minimum no one can justifiably attribute a much simpler structure to them. These are cells that have only grown out in two opposite directions and contract along their longitudinal axes upon applying a stimulus. But we know of other cells that branch out into numerous extensions that all contract in response to stimuli, and can even be retracted in such a way that a cell that might previously be extensively branched and ramified now appears as a rounded lump.^FF^ We are familiar with these cells in chameleons, for example, where their projections are all pointed at the surface of the skin; or in frogs, where the projections spread out parallel to the skin surface in every direction, and it is likely that these articulated cells can be found in all color changing amphibians (Fig. 8).^GG^ What right do we have to assume that the contractile substance, which pervades these cell bodies in every direction out to their most distant extensions, is more simply built than the contractile contents of the muscle cells? It is possible that it is built much differently, but thus far we are completely unable to say whether it is simpler or more complicated.

We still have not yet explored any kind of contractile substance enough to be aware of any connection between its structure and its physiological properties.

In the pigment cells it is possible that these movements could easily observed because the pigment would be conspicuously distinguished from its surroundings. Many of these kinds of cellular movement have certainly been overlooked because this expedient is lacking, yet nevertheless there is no shortage of available observations. Cells embedded in tissues have been shown to have movement just as in cells swimming freely in fluid, such as the lymphocytes in vertebrates and the hemocytes in invertebrates.

In many plants it has been shown that what is called the circulation of the cell sap [*Zellsaftströmungen*] is in fact not currents of a free fluid uniformly filling the cavity of the cell, nor is it movements of small molecules in this fluid. Rather, it comes from the movements from the protoplasm—in other words, from the living cellular body.^9^ To me this is as erroneous as the belief that the so-called molecular movement in salivary corpuscles as a movement of small grains within a fluid-filled vesicle.^HH^

I have already mentioned that I have never seen such a vesicle burst and release its contents by compression. Rather, the cell would be squeezed into a flat cake that still contained all of its granules, and the granules lost their movement permanently. This suggests that the granules were components of a small organism that was killed by crushing it, depriving it of its motility.

How complicated must the mechanisms be to make these movements we have discussed? Let us also keep in mind that we have so far considered only the phenomena of movement that are perceptible by means of the microscope. We have thus far considered a range of phenomena that could be compared to movements in larger animals that we can see with the naked eye. We have not yet considered the means by which the small organism feeds itself, grows, and begets its own kind; we have not yet considered the means by which it exerts specific effects, depending on whether it is a nerve cell, a glandular cell, etc.

If we consider all this, we must acknowledge that we are dealing with organisms whose complexity we cannot compare with that of animals, as we have no right to assume that they are again composed of countless small organisms. But we must nevertheless admit that they represent a highly artful structure whose essential architectural elements have been as yet completely hidden from our view. All these elementary organisms, animal and plant, look similar to each other in their first youth, just as the embryos of the individual animals of the zodiac, the vertebrates, the arthropods, the cephalopods, and so on, look more similar to each other than they do when they are fully developed. The observation of this fact was the great discovery that enabled Theodor *Schwann* to illuminate the whole of histology with such a bright light. But with regard to his opinions on whether external similarities might be based on internal structures, here he made essential errors.

The schema—solid cell membrane, initially fluid cellular content, and cell nucleus with nucleoli—has become worthless for us. Indeed, the time has come when clinging to this schema is downright harmful for the further development of histology: for it is for the schema’s sake that membranes are assumed where none have been proven, for the schema’s sake the cell contents are treated as a fluid unless the opposite is shown in specific cases, for the schema’s sake that every cell must have had a nucleus at some point even if one has never been seen, for the schema’s sake that intercellular substances whose development separate from the cells cannot be proven, indeed is highly unlikely, etc.

Since the name “cells” is so closely connected with this schema, I might have even suggested banishing it completely, were it not tied up with such a glorious era of histology. If we freed ourselves from this schema, the elementary organisms could still be called cells, we will still know what this [term] means, and later generations will remember the valiant fighters who have conquered the whole field of histology under the banner of the cell theory.

## Translator’s Annotations

A. Schwann (1839, p. 220ff.). The English translation is Schwann (1847, p. 186ff.).
B. I am translating Brücke’s *Zelleninhalt* as “cell content,” “cell contents,” “cellular content,” etc.; his emphasis on the contents of the cell is reflected in his neologism *Zellenleib* or “cell body.” On Brücke’s term *Zellenmembran*, see note I, below.
C. Ironically, this is exactly how we understand the microvilli of the brush border today.
D. Funke (1855); Kölliker (1856). The term for columnar epithelial cells used by Brücke and his contemporaries was *Cylinderzellen* or *Cylinderepithelium*, and modern German also includes *Säulenepithel*.
E. Brücke (1857) is discussing osmotic swelling experiments, which involved little more than placing dissected tissues or cells in pure water and observing as they absorbed water and swelled.
F. Brettauer and Steinach (1857).
G. Hartnack’s No. 10 objective was a water immersion objective with a 1/16th inch focal length, which provided a 100 × magnification (sans ocular), given a standard 160 mm tube length; it was introduced in 1859 and regarded as one of the best objective lenses of the 1860s (Bradbury 1967, p. 234).
H. Brücke here is deploying a physicalist description of color phenomena as the selective absorption of light wavelengths. His subsequent discussion about a “difference in absorbance” (*Unterschied im Absorptionsvermögen*) and a “noticeable difference in light and color” is, in more modern terminology, simply *contrast* of brightness and color—that is, there must be sufficient contrast between two objects to distinguish them from one another.
I. Brücke consistently uses the word *Membran* to refer to the cell membrane and *Haut* to refer generally to any kind of “skin.” This is unique to Brücke in this essay, as most German biologists in the nineteenth century would have used the terms *Haut*, *Membran*, and *Wand* interchangeably, for both plant and animal cells (Liu 2019). The term *Hülle* was also used, and was usually translated as “envelope.”
J. Carl Heinrich Schultz (1798–1871), Berlin botanist and physician; after 1848 he used the name “Schultz-Schultzenstein,” to distinguish himself from a southern-German botanist with the same name.
K. Schultz (1836) studied frog and salamander blood cells, which are nucleated. Normal mammalian red blood cells lack nuclei.
L. What would later be called “Brownian motion” was discovered by the English botanist Robert Brown (1773–1858) in the summer of 1827 (Brown 1828). Because of the later importance of Brownian motion to Jean Perrin’s proof of the atomicity of matter, the scholarship on this topic in the history of physics is rich (for example, Brush 1968; Nye 1972). However, as far as I know the history of Brownian motion in botany or biology writ large has yet to be written.
M. Schwann (1839, pp. 87–90); in English, Schwann (1847, pp. 77–80).
N. Brücke (1862). These may be the secretory granules of salivary gland cells.
O. The term “vacuole” was coined by Felix Dujardin (1801–1860) to describe membraneless cavities filled with water or other clear fluid, especially in lower organisms such as amoeba and hydra. The term was controversial: at the time, the leading expert on the so-called “infusoria” was Christian Gottfried Ehrenberg (1795–1876), who argued that the *Infusionsthierchen* were “complete” animals, and that the appearance of round holes were in fact stomachs, complete with a muscular lining. However, Dujardin’s term was quickly accepted, and often used in ignorance of its initial, controversial origins. Dujardin (1835), see also Churchill (1989).
P. It is important to note that what we recognize today as the cell membrane, consisting of the lipid bilayer and embedded proteins, was not directly visible until the era of electron microscope: the lipid bilayer cell membrane is about 7–10 nm thick, while a light microscope can theoretically resolve objects no smaller than about 250 nm (Liu 2019). Although Brücke could not have known the true dimensions of the cell membrane as we know it today, his discussion here shows his awareness of the problem of using the microscope to identify surface interfaces vs. more substantial boundaries. For example, an oil droplet in water will have what Brücke calls a single contour line at their surface interface, but this surface does not have a structurally independent existence.
Q. The idea that the plant cell protoplast (to use a more modern term) secretes the cellulosic membrane was established in 1844 by Hugo Mohl (1805–1872) and accepted by almost all botanists. Botanists briefly debated whether it was possible for a plant cell to exist without a cell wall, and whether the protoplast had its own inner and outer membranes. There is no sign that Brücke was aware of these botanical debates.
R. Schwann had argued that cytoblastema and thus free cell formation could occur either within preexisting cells or outside of them. As Brücke demonstrates here in “The Elementary Organisms” endogenous free cell formation was still accepted in well into the 1860s—in reproductive cells in plants (Farley 1982, pp. 88–100), and in pathological growths in animals (Harris 1999, Chap. 13).
S. Compare Brücke’s description here to Lenoir (1983, Chap. 5).
T. The cellular nature of cartilage had been a centerpiece of Schwann’s *Microscopic Investigations* in 1838/39 (Duchesneau 1987, pp. 170–175).
U. At issue was whether (a) the cartilage cells are formed from the cartilage intercellular substance, as would be true in Schwann, C. B. Reichert, and Rudolf Virchow’s theory of intercellular substances; or (b) the “intercellular” substance of the cartilage is formed by the cartilage cells, as would be the case in the theory Brücke’s presents here, i.e., that cells are the sole agents of this kind of tissue growth. This passage is confusing to a modern reader because Brücke is describing connective tissue growth as a process of metamorphosis of the cell’s body, or at least its outer reaches. Today we would consider these tissues to be kinds of extracellular matrix that are secreted by cells.
V. Alexander Rollett (1834–1903), Brücke’s assistant in Vienna from 1858 to 1863, and professor of histology and physiology at the University of Graz from 1863 to his death.
W. Virchow (1851) demonstrated that bone, cartilage, and connective tissue had homologous development and cellular structure, all consisting of intercellular substance and morphologically identical cells—the *Knochenkörperchen, Knorpelkörperchen*, and *Bindegewebskörperchen*, or in current terminology the osteocytes, chondrocytes, and (perhaps) the mesenchymal stem cells. The Brückean school’s views were summarized by Rollett, in Stricker (1870, Chap. 2).
X. It is not clear to me whether Brücke meant his neologism *productionsfähige Zelle* or “cell capable of production” to be synonymous with “a cell capable of producing another” alone, or if he meant something broader. At other points in the essay Brücke used more typical terms for reproduction, *Fortpflanzung* and *Erzeugung*. Müller-Wille (2010); Vienne (2017); Hopwood et al. (2018, Chap. 20).
Y. In early Linnean taxonomy the Cryptogamia were plants whose sexual organs were hidden or nonexistent and which did not produce seeds, including the algae, lichens, liverworts, mosses, fungi, and ferns. The Phanerogamia (spermatophytes in modern terminology) were seed-bearing plants with differentiated, visible sexual organs. See Farley (1982).
Z. On the history of theories of direct vs. indirect nuclear division, see Harris (1999, Chap. 14) and Churchill (2015, pp. 243–260).
AA. Eduard-Gérard Balbiani (1823–1899), well known Italian-German-French-Haitian-Creole biologist.
BB. On the history of Hugo Mohl’s primordial utricle theory see Liu (2017).
CC. In what follows Brücke discusses the consistency (*Consistenz*) of protoplasm and the nucleus, as being either low or high. I have substituted “soft” and “firm” for low/high consistency; however, it is clear that Brücke had in mind the degrees of consistency or cohesion of an aggregate substance, not just its feel or texture.
DD. Brücke (1858). Brücke wrote a clearer summary in Stricker (1870, Chap. 6).
EE. Margo (1860).
FF. These are the chromatophores.
GG. Brücke (1852).
HH. Brücke (1862).

## Note on the Translation, and Acknowledgments

I initially attempted this translation in August 2023 as a test of DeepL, a machine translator based on a convolutional neural network and trained on the Linguee bilingual concordance database. However, because Linguee’s concordance database is built with web crawlers, it is less suitable for translations of historical sources. The machine translation nevertheless worked as a framework for my own: since I am a novice translator and did not use a computer-assisted translation software package like Trados or OmegaT, the DeepL translation was helpful for me to get a grip on the text on a sentence-by-sentence basis.

My thanks to Mathias Grote for his careful check of the translation. Karl Matlin provided invaluable help on current terminology and perspectives on the histological structures Brücke described; translating the text would have been impossible if I were not able to translate the biology as well.

As indicated in the notes, two translated passages are not my own. Brücke quoted a long passage from Theodor Schwann’s *Mikroskopische Untersuchungen* (1839) and a short one from Carl Nägeli’s “Zellenkerne, Zellenbildung und Zellenwachsthum bei den Pflanzen” (1844), both of which were translated into English shortly after they were published.

## Footnotes

1 I am calling the cells elementary organisms, like we call the elements those bodies that thus far have not been chemically decomposed. As the indivisibility of the elements is unproven, so we cannot deny the possibility that the cells themselves are not perhaps composed of other, even smaller organisms, which stand in a similar relationship to the cells as the cells do to the whole organism; but so far we have no reason to believe this.

2 Plate 21, Fig. 3 from Czermak and Siebold (1850).

3 Schultze (1861).

4 Schwann (1839; pp. 75–76); in English, Schwann (1847; pp. 67–68).

5 Raschkow (1835).

6 Schwann (1839, p. 114 [in English, Schwann 1847, pp. 24–25, 97]).

7 By endogenous cell formation I understand exclusively what botanists called *free cell formation*: formation of daughter cells in the body of the mother cells, as it is observed in the megaspore [*Embryocask*]. I note this because some have lumped cell formation by division together with endogenous cell formation. For me the essential difference lies in the fact that in the one case cells arise and grow like embryos in the mother’s womb, while in the other case the womb of the mother cell disintegrates into pieces that now represent the second generation.

8 Nägeli (1844 [in English, 1846]) says: “Structures resembling nuclei may be detected here and there in the cells of Fungi. The fermentation fungus [*Gährungspilz*] in the must of wine and in beer yeast often exhibits a little nucleus of whitish mucus, lying on the membrane, regularly in each cell.” I will not claim that Nägeli did not see real nuclei in front of him, but nevertheless I can state two things with great certainty: first, that the fungi I examined were in full vitality and abundantly provided with buds [*Sprossen*] of various sizes, and second, that I am equipped with more perfect means of magnification than the famous botanist could have had in 1844. I could not make any nuclei visible, even with iodine tincture, and neither with acetic acid. I think nobody is justified calling these granules of various size and number nuclei.

9 It seems to me that, even now, the so-called circulation of the cell sap is not correctly understood for all cases. So far, I have studied only one object in such detail that I allow myself to make conclusions about it, the stinging hairs of nettles. First of all, I cannot recognize what has been called the currents of the protoplasm. I find the entire inner surface covered with a layer of protoplasm, and the so-called little currents appear to me as strip-like or bead-like protrusions of this protoplasmic layer. Perhaps there are also strands which pass freely through the central cavity, but I have not yet been able to prove this to myself. As far as the movement is concerned, two types can be clearly distinguished: a slow, pulling or creeping one, which depends on changes in the arrangement of the protoplasmic masses; and a second, faster, flowing one, which is perceived via the movement of the numerous granules that are found in the entire protoplasmic mass. It is usually presented as if the whole protoplasmic mass is in a flowing motion and the granules are just dragged along, passively; however, I must firmly deny this in light of my study. There is certainly the appearance of such a flowing movement, but this is partly produced by protoplasm’s contraction movements, like those in muscles, and partly from the granules that are not easily seen. Since these [granules] are not themselves visible, they produce, if I may express myself in this way, small faint nebulae [*Nebelflecke*] that move in the direction of the current, so that it appears as if the whole protoplasm is in a flowing movement. I believe this is in fact not the case for the following reasons: First, one sees globules flowing in opposite directions and vibrating around each other, often in very narrow paths (the so-called currents). This would be quite impossible if one were dealing with the flow of a viscous liquid, as is usually stated. Second, the movements of the protoplasm can be distinguished quite clearly from the movements of the granules. For this purpose it is best to position the basal part of the cell at a short distance from the group of cells in which it is implanted, then at high magnification (Hartnack immersion system No. 10, ocular 3) adjust so that the central plane is clearly visible and a cross section of the protoplasmic mass comes into view. With sustained observation, it is clearly visible how the protoplasmic mass drives bulge-like protrusions towards the interior, which change their shape for a while and finally disappear again. The movement of the granules continues independently. The so-called protoplasm appears as the contractile cellular body, which encloses an irregular cavity at the base due to its strip- and bulge-like protrusions and which has fluid flowing through it that contains numerous small granules. Obviously there is some comparison to be made between this fluid and the blood in an animal body; but such an analogy is worthless as long as we do not know more than what we know now about the structure and the economy of the cellular body.
